# Efficient TiO_2_ Surface Treatment Using Cs_2_CO_3_ for Solution-Processed Planar-Type Sb_2_S_3_ Solar Cells

**DOI:** 10.1186/s11671-019-2858-5

**Published:** 2019-01-17

**Authors:** Wook Hyun Kim, Sungho Woo, Kang-Pil Kim, Soo-Min Kwon, Dae-Hwan Kim

**Affiliations:** Convergence Research Center for Solar Energy, Daegu Gyeongbuk Institute of Science and Technology (DGIST), Daegu, 42988 Republic of Korea

**Keywords:** Planar-type Sb_2_S_3_ solar cell, Electron transport layer, Surface treatment, Solution process, Cs_2_CO_3_

## Abstract

We report a highly effective surface treatment method for planar-type Sb_2_S_3_ solar cells by employing a Cs_2_CO_3_-modified compact TiO_2_ (c-TiO_2_) electron transport layer. It is found that surface treatment using a Cs_2_CO_3_ solution can shift the work function of c-TiO_2_ upward and reduce its surface roughness. As a result, compared with the power conversion efficiency of untreated solar cells, that of the treated solar cells with a glass/FTO/c-TiO_2_(/Cs_2_CO_3_)/Sb_2_S_3_/P3HT/Au structure significantly improved from 2.83 to 3.97%. This study demonstrates that the introduction of Cs_2_CO_3_ on a c-TiO_2_ layer is a simple and efficient way to adjust the work function of the electron transport layer and fabricate high-performance planar-type Sb_2_S_3_ solar cells.

## Background

Recently, many inorganic metal chalcogenides based on earth-abundant elements such as copper zinc tin selenide (CZTS), lead sulfide (PbS), copper (I) sulfide (Cu_2_S), tin sulfide (SnS), and antimony sulfide (Sb_2_S_3_) have been investigated as absorber materials in low-cost thin film solar cells in order to replace the mainstream solution-processible absorbers such as copper indium gallium selenide (CIGS) and cadmium telluride (CdTe) [1]. However, the use of CZTS and PbS in the industry has severe drawbacks, because CZTS uses the toxic and harmful hydrazine (N_2_H_4_) and requires the complex control of multi-compound [2] and PbS contains Pb, which is also toxic and hazardous. Other potential materials such as Cu_2_S and SnS have relatively low efficiencies compared to those of CIGS and CdTe. Sb_2_S_3_, however, has attracted attention as a candidate material due to its suitable band gap (~ 1.65 eV) and high absorption coefficient (> 10^5^ cm^−1^) for efficient light absorption, high dielectric constant for exciton dissociation, and good band alignment with various hole transport layers (HTLs) for efficient charge carrier transfer, in addition to its cost effectiveness, low toxicity, and excellent air stability [3–6].

There are two types of Sb_2_S_3_ solar cells based on the device structures: sensitized solar cell or planar-type solar cell. Sensitized solar cells originated from dye-sensitized solar cells (DSSCs) and have a F-doped tin oxide (FTO)/compact TiO_2_ (c-TiO_2_)/mesoporous TiO_2_ (m-TiO_2_)/Sb_2_S_3_/HTL/Au structure, while planar-type solar cells have a FTO/c-TiO_2_/Sb_2_S_3_/HTL/Au structure [7].

In terms of device efficiency, sensitized Sb_2_S_3_ solar cells have a higher value than planar types due to their enhanced light-absorbing interfacial area owing to the m-TiO_2_ structure. The factor that decides the performance of sensitized solar cells is their interface quality inside the device where charge carrier separation and transfer occur. Therefore, significant effort has been devoted to the optimization of the interfacial properties, including those of the m-TiO_2_/Sb_2_S_3_ interface, Sb_2_S_3_/HTL interface, and HTL material itself [8]. Various kinds of HTL materials, such as 2,2′,7,7′-tetrakis[N,N-di(4-methoxyphenyl)amine]-9,9′-spirobifluorene (Spiro-OMeTAD) [9]; CuSCN, an inorganic p-type material [10]; poly(3-hexylthiophene) (P3HT), a conducting polymer [11]; and poly(2,6-(4,4-bis-(2-ethylhexyl)-4H-cyclopenta [2,1-b,3,4-b′]dithiophene)-alt-4,7(2,1,3-benzothiadiazole)) (PCPDTBT), a newly developed conjugated polymer [12], have been applied to adjust the Sb_2_S_3_/HTL interface and hole transport properties leading to a high fill factor (FF) and increased short-circuit current density (*J*_SC_).

Several studies that focus on the improvement of the m-TiO_2_/Sb_2_S_3_ interface properties have been also reported. Tsujimoto et al. modified the m-TiO_2_ surface using Mg^2+^, Ba^2+^, and Al^3+^, which effectively increase the power conversion efficiency (PCE) of all inorganic Sb_2_S_3_ solar cells that have the FTO/c-TiO_2_/m-TiO_2_/Sb_2_S_3_/CuSCN/Au structure [13]. Lan et al. used Li-doped m-TiO_2_ to enhance the electron transport properties and change the Fermi energy level [14]. Fukumoto et al. reported the surface treatment of the Sb_2_S_3_/HTL interface using 1-decylphosphonic acid (DPA), which can be attached to both an uncovered m-TiO_2_ surface and Sb_2_S_3_ surface to reduce recombination and increase the open-circuit voltage (*V*_OC_) and FF [15].

In planar-type solar cells, in contrast to sensitized ones, charge carrier transport depends on the carrier mobility and diffusion length within the Sb_2_S_3_ layer, which are strongly correlated with the morphology, grain size, and crystallinity of the layer. Hence, most research on planar-type solar cells has been focused on improving the Sb_2_S_3_ thin film quality to achieve a large grain size and a high crystallinity by using various deposition techniques. For example, conventional chemical bath deposition (CBD) [16], thermal evaporation (TE) [17], rapid thermal evaporation (RTE) [18, 19], atomic layer deposition (ALD) [20], and nanoparticle ink coating [21] have been applied to fabricate Sb_2_S_3_ thin films. Recently, Wang et al. reported a fast chemical approach (FCA) that can be used to generate very large grain sizes via a one-step spin-coating process and subsequent annealing process using a butyldithiocarbamic acid (BDCA)-based metal-organic precursor solution [22]. Many types of metal oxides or hydroxides can be dissolved in BDCA, which is relatively nontoxic, inexpensive, and thermally degradable, and can be easily synthesized via the reaction of 1-butylamine (CH_3_(CH_2_)_3_NH_2_) and carbon disulfide (CS_2_) [23].

Although the sensitized solar cells have a higher PCE (3–7.5%) than planar-type ones (2.5–5.8%), their device structure and fabrication process are complicated. Moreover, they contain a high degree of interface defects. A planar-type Sb_2_S_3_ device would have more potential for use in industrial-scale solar cells with a high efficiency and low cost, because it is conceptually simpler and easier to scale up and it is highly reproducible [24, 25].

Here, we report the surface treatment of a c-TiO_2_ layer using Cs_2_CO_3_ solution to enhance the performance of planar-type Sb_2_S_3_ solar cells. The Sb_2_S_3_ layer was deposited via a simple FCA spin-coating process to realize a large grain size, which was previously reported by Wang et al.

Cs_2_CO_3_ has been widely studied for application in organic photovoltaics (OPV) [26–28], organic light-emitting devices (OLEDs) [29], and perovskite solar cells (PSCs) [30, 31] to improve electron transport due to its low-work function property. Although Cs_2_CO_3_ is usually decomposed at 550–600 °C, Liao et al. reported that Cs_2_CO_3_ can be decomposed into low-work function cesium oxide via a low-temperature (150–170 °C) thermal annealing process [26]. However, to the best of our knowledge, there is no study on the application of Cs_2_CO_3_ to Sb_2_S_3_ solar cells.

Surface treatment using Cs_2_CO_3_ can not only reduce the energy barrier by changing the work function of c-TiO_2_, but also reduce the series resistance of the device by reducing the surface roughness of c-TiO_2_. The treatment resulted in improved device parameters such as the *V*_OC_, *J*_SC_, and FF, and the PCE increased from 2.83 to 3.97%. We believe that this surface treatment of c-TiO_2_ using Cs_2_CO_3_ solution can provide a simple and effective way of improving device performance in planar-type inorganic metal chalcogenide solar cells.

## Methods/Experimental

### Materials Used and Synthesis of Sb Complex

Antimony (III) oxide (Sb_2_O_3_, 99.99%), CS_2_ (> 99.9%), n-butylamine (CH_3_(CH_2_)_3_NH_2_, n-BA, 99.5%), cesium carbonate (Cs_2_CO_3_, 99.9%), 2-methoxyethanol (CH_3_OCH_2_CH_2_OH, 99.8%), titanium (IV) isopropoxide (Ti(OCH(CH_3_)_2_)_4_, TTIP, 97%), poly(3-hexylthiophene) (P3HT, Mw 50–70K, regioregularity 91–94%, Rieke Metals), 1,2-dichlorobenzene (o-DCB, 99%), and ethanol (CH_3_CH_2_OH, anhydrous) were purchased from Sigma-Aldrich Co. and were used as received without further purification.

The Sb complex was synthesized according to a reported method [22]. Sb_2_O_3_ (1.0 mmol) was mixed with a solution of ethanol (2.0 mL) and CS_2_ (1.5 mL) with magnetic stirring at room temperature. Then, n-butylamine (2.0 mL) was added to the solution slowly under continued stirring for at least 30 min to obtain a homogenous solution of antimony butyldithiocarbamates (Sb(S_2_CNHC_4_H_9_)_3_). Afterwards, 2 mL of this solution was diluted with 1 mL ethanol to form the Sb complex.

### Device Fabrication

The planar-type Sb_2_S_3_ solar cells in this study have a typical structure of FTO/c-TiO_2_/Sb_2_S_3_/P3HT/Au, where P3HT is employed as the HTL. The c-TiO_2_ layer was deposited onto a cleaned FTO surface by spin-coating a mixed solution of 2 mL TTIP, 60 mL ethanol, 0.225 mL distilled water, and 0.03 mL HNO_3_ at 3000 rpm for 30 s, followed by annealing at 500 °C for 60 min in air.

For surface modification using Cs_2_CO_3_, Cs_2_CO_3_ dissolved in a CH_3_OCH_2_CH_2_OH solution with certain concentrations (1, 3, 5, and 10 mg/mL) was spin-coated on a 10-min UV-ozone treated c-TiO_2_ layer at 6000 rpm for 45 s. The films were then heat-treated at 150 °C for 10 min before the Sb_2_S_3_ layer was spin-coated.

For the Sb_2_S_3_ thin films, the Sb complex solution was spin-coated at a speed of 6000 rpm for 30 s, after which the films were annealed on a N_2_-purged hot plate at 200 °C for 1 min and 350 °C for 2 min.

P3HT solution (10 mg in 1 mL o-DCB) was spin-coated on the Sb_2_S_3_/c-TiO_2_/FTO substrate at a speed of 3000 rpm for 60 s, which was then heated on a hot plate at 100 °C for 30 min in air. Finally, the Au counter electrode was deposited using a thermal evaporator under a pressure of 5.0 × 10^−6^ Torr. Each device had an active area of 0.16 cm^2^.

### Measurement and Analysis

The surface and cross-sections of the Sb_2_S_3_ thin films were characterized using field-emission scanning electron microscopy (FE-SEM, S-4800, Hitachi). The surface morphology was studied using atomic force microscopy (AFM, Park NX10, Park Systems). The optical properties of c-TiO_2_ were determined using a UV-Vis (Lambda 750, Perkin Elmer). The current density–voltage (*J*–*V*) characteristics were determined using a specialized solar cell measurement system equipped with an electrometer (model 2400, Keithley) and solar simulator (91192, Newport) with a 1-kW Xenon arc lamp (Oriel). The light intensity was adjusted to one sun (100 mW/cm^2^) under AM 1.5G solar irradiation conditions using a radiant power energy meter (model 70260, Oriel). The series resistance (*R*_S_) and shunt resistance (*R*_SH_) were calculated from the slope of the corresponding *J*–*V* curves beyond *V*_OC_ and *J*_SC_, respectively. The external quantum efficiency (EQE) was measured by a QuantX-300 quantum efficiency measurement system (Newport) equipped with a 100 W Xenon lamp. The structural information of FTO/c-TiO_2_(/Cs_2_CO_3_) sample was characterized by multi-purpose X-ray diffraction (XRD) system (Empyrean, PANalytical) with *θ*-2*θ* mode at a scan rate of 0.05°/sec. The electronic state and energy level were analyzed using X-ray photoelectron spectroscopy (XPS) and ultraviolet photoelectron spectroscopy (UPS) in an ultrahigh vacuum environment (ESCALAB 250Xi, Thermo Scientific). UPS and XPS spectra were obtained by using the He I line (hν = 21.2 eV) and the Al Kα radiation source (hν = 1486.6 eV), respectively. The XPS depth profiling was obtained using Ar^+^-cluster ion gun and etch rate of 1 Å/sec.

## Results and Discussion

**Fig. 1:**
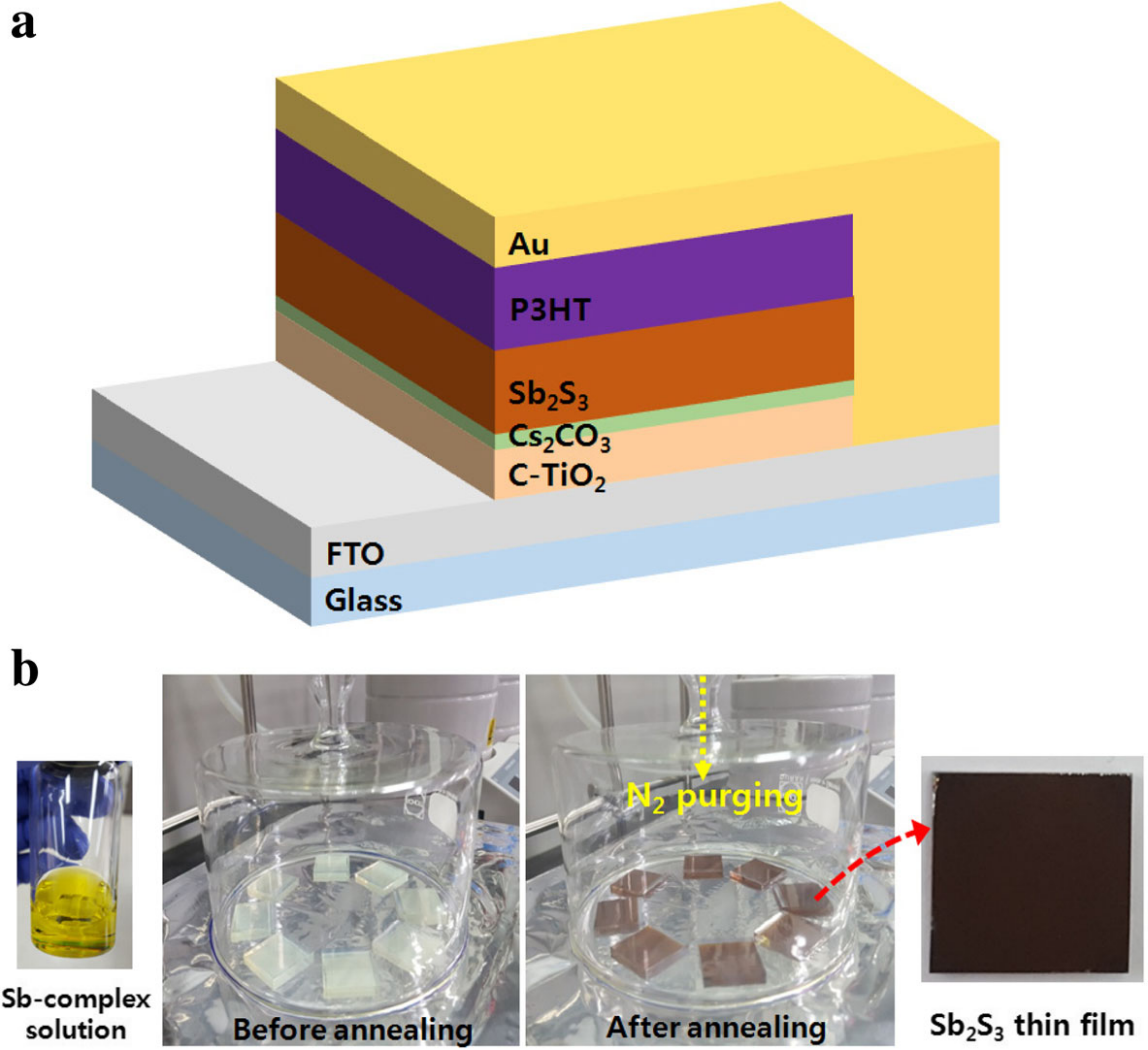
**a** Schematic of the device structure of planar-type Sb_2_S_3_ solar cells. **b** Sb_2_S_3_ thin film fabrication process using FCA method

Figure 1a shows a schematic of the device structure. The bottom layer is composed of c-TiO_2_ layers on a glass/FTO substrate acting as electron transporting. Light is absorbed by the Sb_2_S_3_ layer, while holes are transported by the P3HT HTL and collected at the Au counter electrode.

**Fig. 2:**
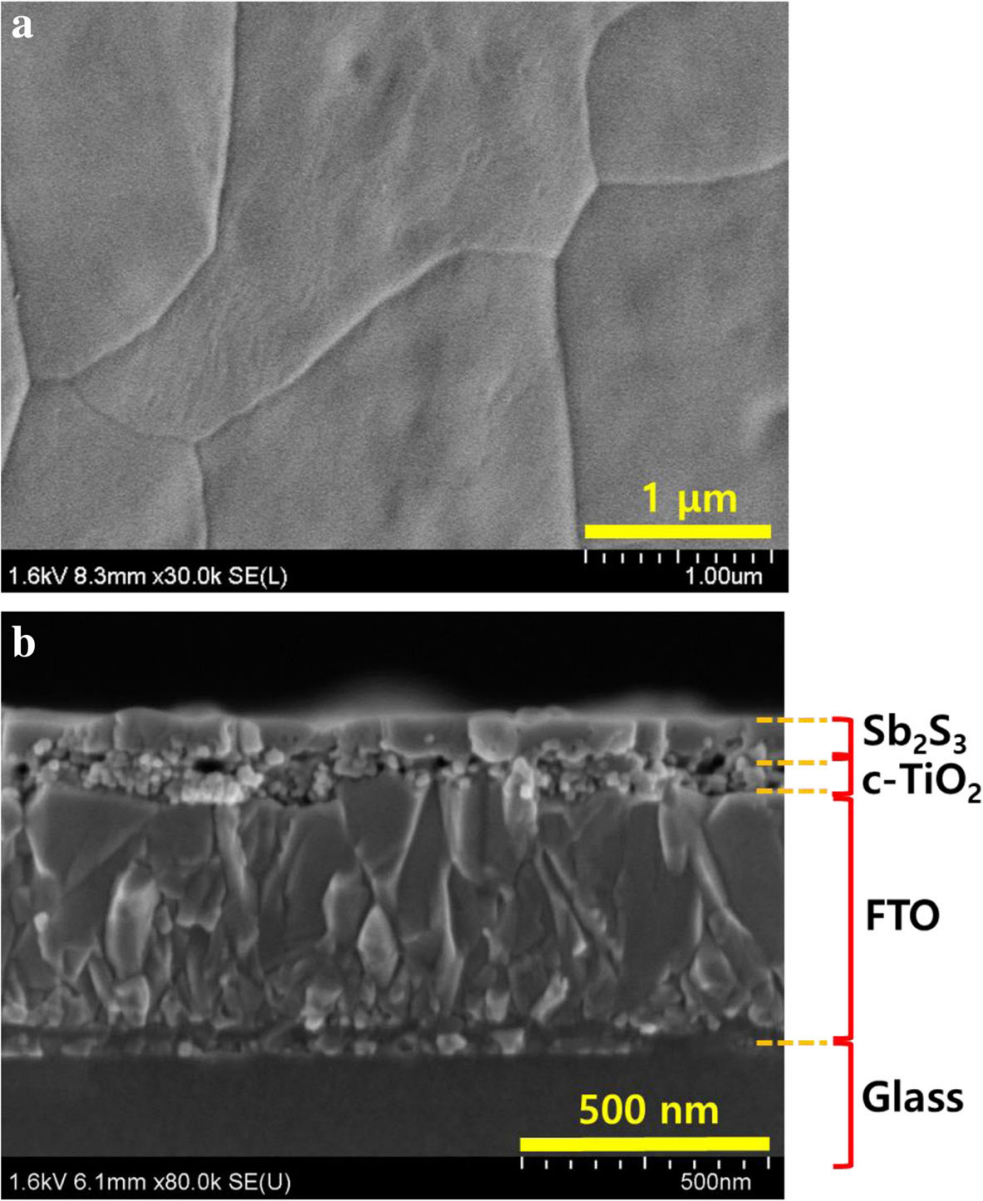
**a** Top view and **b** cross-sectional SEM images of Sb_2_S_3_ absorbing layer after annealing at 350 °C for 2 min

The Sb_2_S_3_ absorbing layer was deposited via the FCA using the Sb complex precursor to realize very large grain sizes. The precursor was thermally decomposed to the amorphous state at 200 °C for 1 min and crystalline state at 350 °C for 2 min (Fig. 1b). The SEM image shown in Fig. 2 indicates a very large grain size, which is almost the same as the Sb_2_S_3_ thin film morphology reported by Wang et al. [22].

The efficiency of the planar-type Sb_2_S_3_ solar cell was improved via surface treatment with Cs_2_CO_3_ of the c-TiO_2_ layer.

**Fig. 3:**
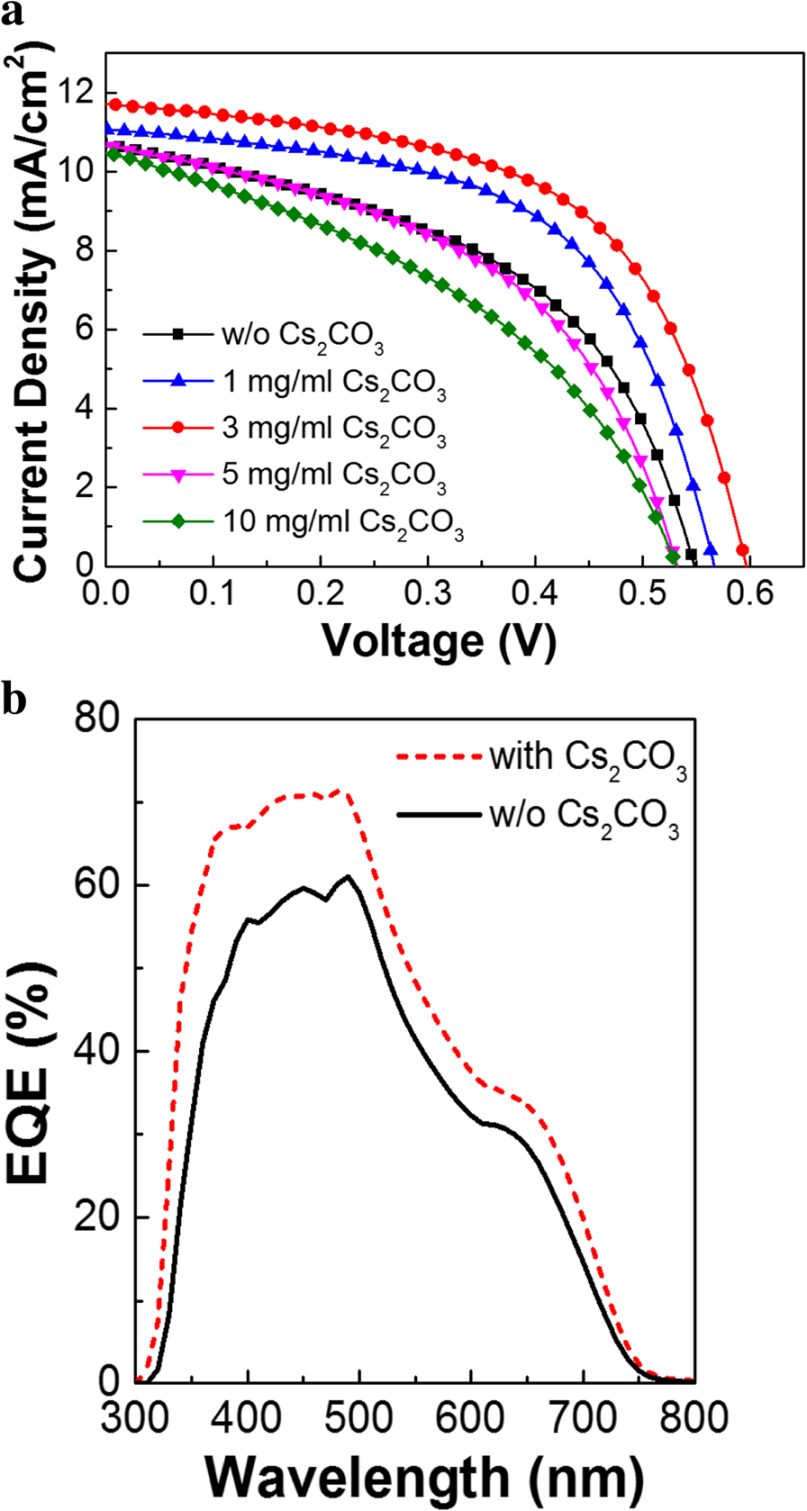
**a** Current density–voltage (*J*–*V*) characteristics and **b** EQE spectra of planar-type Sb_2_S_3_ solar cells with and without Cs_2_CO_3_ treatment of c-TiO_2_

The device properties based on the concentration of Cs_2_CO_3_ solution were performed to determine the optimum Cs_2_CO_3_ concentration. Figure 3a and Table 1 show the *J*–*V* characteristics for the devices using different concentrations of Cs_2_CO_3_ solution under AM 1.5G illumination (100 mW/cm^2^). When the concentration is too low (1 mg/mL), there is a problem in whole coverage of the c-TiO_2_ surface with Cs_2_CO_3_. However, if it is too high (5 and 10 mg/mL), it acts as a dielectric material, resulting in an increase in the series resistance and decrease in the device efficiency. The optimum concentration of Cs_2_CO_3_ was found to be 3 mg/mL. (Hereafter, “with Cs_2_CO_3_ treatment” means treatment using 3 mg/mL concentration of Cs_2_CO_3_ unless otherwise noted.)

**Table 1:** Summary of device performances according to different Cs_2_CO_3_ concentrations under AM 1.5G condition

Devices	*V*_OC_ (V)	FF (%)	*J*_SC_ (mA/cm^2^)	PCE (%)	*R*_S_ (*Ω* cm^2^)	*R*_SH_ (*Ω* cm^2^)
Without Cs_2_CO_3_	0.549	48.14	10.71	2.83	11.14	178.56
With 1 mg/mL Cs_2_CO_3_	0.567	56.82	11.07	3.56	9.42	451.2
With 3 mg/mL Cs_2_CO_3_	0.596	56.89	11.71	3.97	8.82	454.08
With 5 mg/mL Cs_2_CO_3_	0.532	47.99	10.66	2.72	10.66	207.36
With 10 mg/mL Cs_2_CO_3_	0.531	40.78	10.50	2.27	15.17	125.76

As a result, the device had a PCE of 2.83%, *V*_OC_ of 0.549 V, *J*_SC_ of 10.71 mA/cm^2^, and FF of 48.14% before the treatment. However, after the treatment with 3 mg/mL solution, all these parameters increased significantly, i.e., to a *V*_OC_ of 0.596 V, *J*_SC_ of 11.71 mA/cm^2^, and FF of 56.89%, leading to a PCE of 3.97%. This treatment resulted in a ~ 40% improvement in the PCE. The higher EQE over full spectrum range as shown in Fig. 3b indicates that the light is more efficiently converted into current leading to increase in *J*_SC_ by this Cs_2_CO_3_ treatment. From the EQE spectra, we can also see that the onset of EQE at 750 nm corresponds well to a band gap of 1.65 eV for Sb_2_S_3_ layer and a decrease in EQE from 500 to 650 nm is attributed to the absorption of P3HT HTL layer.

**Fig. 4:**
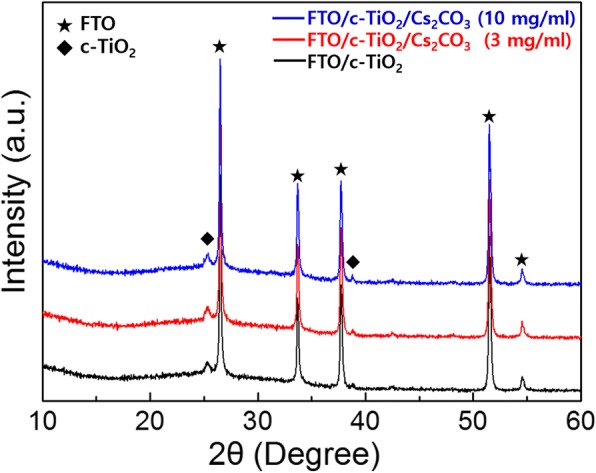
XRD patterns of the c-TiO_2_ on FTO glass substrates with and without Cs_2_CO_3_ treatment

**Fig. 5:**
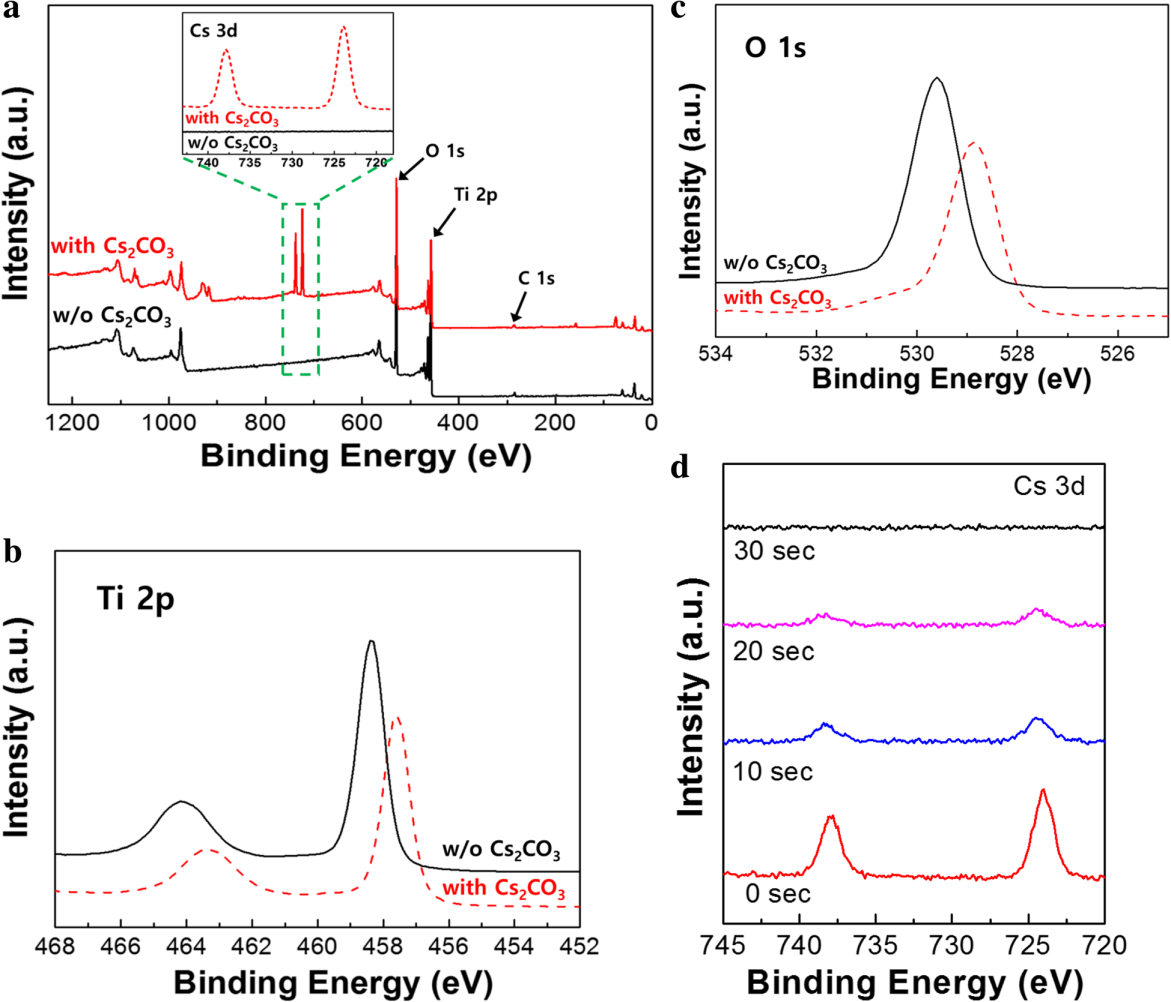
XPS spectra of **a** survey scan and Cs 3d peak, **b** Ti 2p peak, **c** O 1 s peak for c-TiO_2_ surface with and without Cs_2_CO_3_ treatment, and **d** depth profile for Cs 3d peak for FTO/c-TiO_2_/Cs_2_CO_3_ sample to determine the thickness of Cs-related layer

**Fig. 6:**
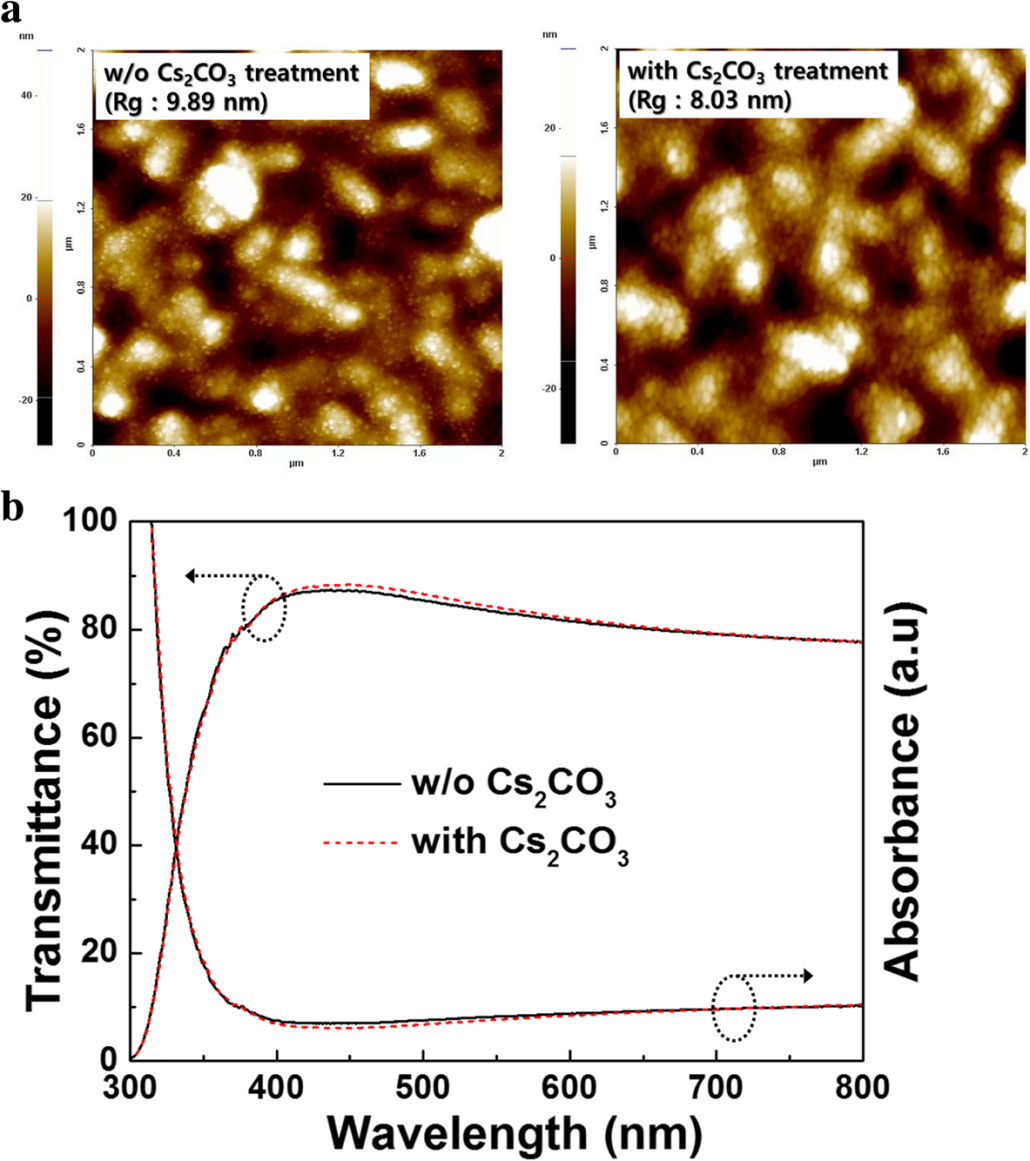
**a** AFM images (2 μm × 2 μm) of the surface morphology and **b** UV-Vis absorption and transmission spectra of c-TiO_2_ with and without Cs_2_CO_3_ treatment

We measured the XRD patterns of the c-TiO_2_ on FTO glass substrates with and without Cs_2_CO_3_ treatment to investigate whether Cs_2_CO_3_ has effects on the crystallization of the c-TiO_2_ layer and/or the formation of new secondary phase by diffused Cs-related species. There was no change in the XRD peak after Cs_2_CO_3_ treatment as shown in Fig. 4. This indicates that the Cs_2_CO_3_ treatment has little effect on the crystal structure of c-TiO_2_ and also does not create a new phase. Furthermore, there was no evidence of a decomposed Cs-related phase (cesium oxide, cesium suboxide, or Cs element) after thermal treatment of Cs_2_CO_3_, which means that the thickness of the Cs_2_CO_3_ is very thin. As shown in Fig. 5d, the thickness of Cs-related species was about 2~3 nm, which was determined by XPS depth profile analysis for the sample of FTO/c-TiO_2_/Cs_2_CO_3_ (3 mg/mL). The measured thickness of Cs_2_CO_3_ (2~3 nm) is in good agreement with the AFM analysis, which shows improved surface roughness through Cs_2_CO_3_ treatment from 9.89 to 8.03 nm (see Fig. 6a).

We studied the surface state of the c-TiO_2_ layer using XPS measurements. The XPS spectra in Fig. 5 show that both the survey scan and Cs 3d peak scan clearly indicate the existence of Cs on the c-TiO_2_ surface. The Ti 2p and O 1 s peaks were shifted to lower binding energies owing to the Cs_2_CO_3_ treatment, which indicates that the Cs_2_CO_3_ treatment affected the electronic structure of the c-TiO_2_ layer. The appearance of a slight shoulder at ~ 531 eV in the O 1 s spectrum could be attributed to the cesium oxide generated from Cs_2_CO_3_ decomposition via annealing at 150 °C, which has a low work function [26].

The AFM images in Fig. 6a reveal a difference in the surface morphology of the c-TiO_2_ layer before and after Cs_2_CO_3_ treatment. The surface became smoother and the root mean square roughness (Rg) decreased from 9.89 to 8.03 nm after treatment. This smooth surface was useful for increasing the physical contact between the c-TiO_2_(/Cs_2_CO_3_) layer and the Sb_2_S_3_ layer, leading to a decrease in the *R*_S_ value from 11.14 Ω cm^2^ (without Cs_2_CO_3_) to 8.82 Ω cm^2^ (with Cs_2_CO_3_) (see Table 1). The decreased *R*_S_ may have contributed to increasing the FF from 48.14 to 56.89% [5].

The UV-Vis transmittance spectra of the c-TiO_2_ films with and without Cs_2_CO_3_ are shown in Fig. 6b. The figure shows that there is little change in the optical transmittance between wavelengths of 300 and 800 nm, which confirms that Cs_2_CO_3_ treatment has a negligible effect on the intensity of light reaching the Sb_2_S_3_ layer.

**Fig. 7:**
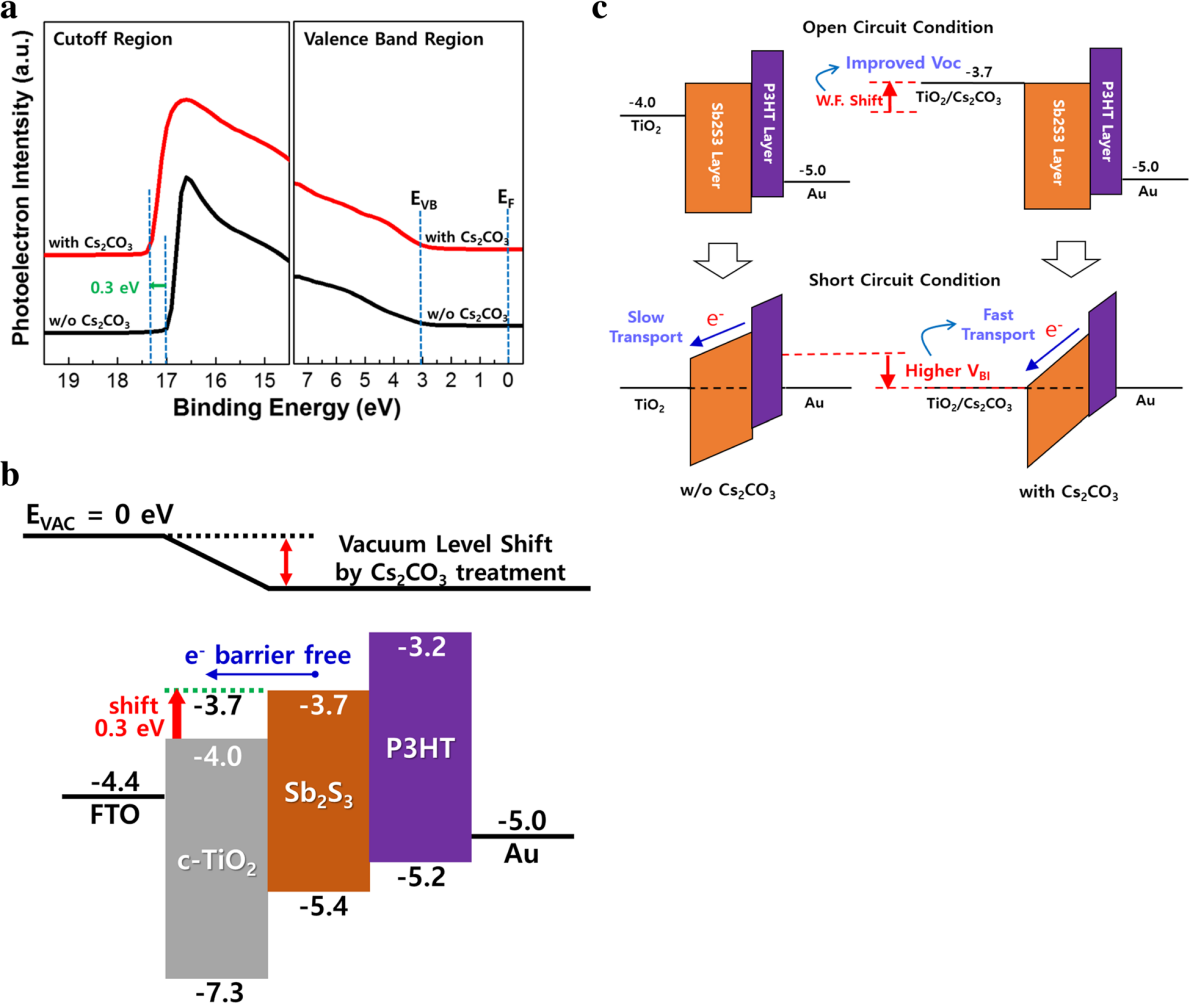
**a** UPS spectra of c-TiO_2_, **b** energy level diagram, and **c** proposed operating principle of planar-type Sb_2_S_3_ solar cells with and without Cs_2_CO_3_ treatment

UPS was used to determine the change in the work function of the c-TiO_2_ layer before and after Cs_2_CO_3_ treatment to investigate the effect of Cs_2_CO_3_ on *V*_OC_. The results are shown in Fig. 7a. The work function of c-TiO_2_ decreases by 0.3 eV after Cs_2_CO_3_ treatment. Cs_2_CO_3_ is widely used as an efficient electron transport material in many optoelectronic devices through thermal evaporation or solution process. However, the accurate analysis of electron transport mechanism and the type of decomposed Cs-related species that are responsible for electron transport property are still uncertain and controversial. Among previous reports on solution-processed Cs_2_CO_3_, Liao et al. showed that Cs_2_CO_3_ can be decomposed into low work function, doped semiconductor in the form of Cs_2_O doped with Cs_2_O_2_ after thermal annealing at 150 °C by using XPS analysis [26]. This form of doped cesium oxide can act as an n-type semiconductor with intrinsically low work function, which might contribute to work function reduction of c-TiO_2_ in our system. In addition, there was no change in the absorption onset as shown in Fig. 6b, indicating little change in the optical bandgap of the c-TiO_2_ after the treatment.

The energy band diagram in Fig. 7b shows that the conduction band energy level of c-TiO_2_ shifted toward a lower energy by 0.3 eV. This shift leads to not only an improved *V*_OC_ due to an increase in the built-in potential (*V*_BI_) inside the devices, but also an increased *J*_SC_ due to the alignment of the energy level between c-TiO_2_ and Sb_2_S_3_ to reduce the charge transport barrier at the interface. The proposed operating principle is illustrated in Fig. 7c. At open-circuit condition, the shifted conduction band of the c-TiO_2_ layer by Cs_2_CO_3_ treatment leads to the increased V_BI_, which contributes to the improved *V*_OC_. At the same time, the increased V_BI_ results in the larger energy band bending of the Sb_2_S_3_ layer under short-circuit conditions, and thus the photogenerated electrons can move quickly toward the c-TiO_2_ layer. This fast electron transport is attributed to cause the enhanced *J*_SC_ and FF. Thus, the Cs_2_CO_3_ treatment on c-TiO_2_ layer could increase both *V*_OC_ and *J*_SC_ simultaneously, leading to the enhanced PCE. Hence, Cs_2_CO_3_ is a promising material for c-TiO_2_ surface modification as it enhances device performance by changing the work function and improving the electron transport properties.

## Conclusions

Cs_2_CO_3_ was found to be an effective surface modifier to enhance the charge transport ability of the c-TiO_2_ electron transport layer (ETL) for planar-type Sb_2_S_3_ solar cells. The UPS data show that Cs_2_CO_3_ treatment can shift the work function of c-TiO_2_ upward, possibly increasing the built-in potential of the device and reducing the energy barrier for charge transport. The c-TiO_2_ surface became smoother after Cs_2_CO_3_ treatment, resulting in increased physical contact with the Sb_2_S_3_ absorber. The solar cell performance was significantly improved in all parameters simultaneously including *V*_OC_, *J*_SC_, and FF. This resulted in an increase in the PCE from 2.83 to 3.97%, almost a 40% increase. This study shows that surface treatment using inorganic compounds such as Cs_2_CO_3_ will play an important role in the development of highly efficient planar-type Sb_2_S_3_ solar cells.

## Abbreviations

AFM: Atomic force microscopy
c-TiO_2_: Compact TiO_2_
EQE: External quantum efficiency
ETLs: Electron transport layers
FCA: Fast chemical approach
FF: Fill factor
FTO: Fluorine-doped tin oxide
HTLs: Hole transport layers
*J*_SC_: Short-circuit current density
*J*–*V*: Current density–voltage
m-TiO_2_: Mesoporous TiO_2_
P3HT: Poly(3-hexylthiophene)
PCE: Power conversion efficiency
*R*_S_: Series resistance
*R*_SH_: Shunt resistance
SEM: Scanning electron microscopy
UPS: Ultraviolet photoelectron spectroscopy
UV-Vis: Ultraviolet–visible spectrometer
*V*_BI_: Built-in potential
*V*_OC_: Open-circuit voltage
XPS: X-ray photoelectron spectroscopy
XRD: X-ray diffraction
